# Aspirin for Prevention of Cardiovascular Disease

**DOI:** 10.5888/pcd14.170171

**Published:** 2017-09-07

**Authors:** Vincent L. Mendy, Rodolfo Vargas, Lei Zhang

**Affiliations:** 1 Office of Health Data and Research, Mississippi State Department of Health, Jackson, Mississippi

## Abstract

We used data from the 2013 Mississippi Behavioral Risk Factor Surveillance System to examine aspirin use for the prevention of primary and secondary cardiovascular disease (CVD), based on the 2009 US Preventive Services Task Force (USPSTF) guidelines, among Mississippi men (aged 45–79 y) and women (aged 55–79 y) and to explore differences in aspirin use by sociodemographic characteristics. Among those without CVD, 39.1% of men and 45.9% of women reported taking aspirin, and among those with CVD, 85.9% of men and 85.1% of women reported taking aspirin. Data on preventive use of aspirin by sociodemographic characteristics yielded mixed results.

## Objective

Cardiovascular disease (CVD) is the leading cause of death in Mississippi; in 2013 the state’s CVD death rate was 1.4 times the national rate (1). The benefits of aspirin use in CVD prevention have been documented (2–4). The Mississippi State Department of Health (MSDH) and the Centers for Disease Control and Prevention (CDC), through a cooperative agreement (Mississippi Delta Health Collaborative), are implementing interventions across the 18-county Mississippi Delta region. These interventions target the ABCS (aspirin for those eligible, blood pressure control, cholesterol management, and smoking cessation) of heart disease and stroke prevention. However, the prevalence of aspirin use among eligible Mississippians for primary and secondary CVD prevention as recommended by the US Preventive Services Task Force (USPSTF) (5) has not been assessed.

## Methods

The Behavioral Risk Factor Surveillance System (BRFSS) is a state-based, random-digit–dialed telephone survey of the noninstitutionalized US civilian population aged 18 years or older. BRFSS is conducted in all 50 states, the District of Columbia, and 3 US territories (Puerto Rico, Guam, and the US Virgin Islands) and has been approved by the human research review board at each state’s department of health. Detailed information about BRFSS is available (www.cdc.gov/brfss/). Our analyses were restricted to men aged 45 to 79 years and women aged 55 to 79 years who responded to the 2013 Mississippi BRFSS. Guidelines for aspirin use were based on the 2009 USPSTF recommendations (5), not the recommendations issued in 2016 (6), because the 2009 recommendations were current when data were collected in 2013. We excluded respondents who self-reported a contraindication to aspirin use (n = 689) — those who responded to the question “Do you have a problem or health condition that makes taking aspirin unsafe for you?” with either “yes, not stomach related” or “yes, stomach problems” (7). Men aged 45 to 79 and women aged 55 to 79 without CVD or with contraindications to aspirin use were considered eligible to use aspirin for the primary prevention of CVD, and women and men in the same age categories with a history of CVD but without contraindications were considered eligible for aspirin use for secondary prevention.

### Cardiovascular disease

CVD was defined as a yes response to at least one of the following questions: “Has a doctor, nurse, or other health professional ever told you that you had any of the following? 1) a heart attack, also called a myocardial infarction, 2) angina or coronary heart disease, 3) a stroke.” Respondents who had never been told they had CVD were considered eligible for primary prevention, whereas those with a history of CVD were considered eligible for secondary prevention. Analyses were restricted to respondents who self-identified as black or white; these 2 racial groups accounted for 96.6% of the study population. This study was deemed exempt from approval by the MSDH institutional review board. Weighted prevalence and 95% confidence intervals (CIs) were calculated, and the χ^2 ^test was used to examine sociodemographic differences. SAS version 9.4 (SAS Institute Inc) was used to perform all statistical analyses; *P* values of less than .05 were considered significant.

## Results

More than two-thirds (68.0%; 95% CI, 65.2%–71.2%) of eligible adults were white. Most had health insurance, more than a third were obese (body mass index [kg/m^2^] ≥30.0), and 18.9% (95% CI, 16.3%–21.4%) of men and 17.8% (95% CI, 15.4%–20.2%) of women reported having CVD. Among those without CVD, 39.1% (95% CI, 35.4%–42.9%) of men and 45.9% (95% CI, 42.3%–49.4%) of women were taking aspirin for primary prevention.

The prevalence of aspirin use for primary prevention among men was significantly higher among those with health insurance (41.2%; 95% CI, 37.1%–45.4%; *P* = .02), those with diabetes (56.0%; 95% CI, 46.4%–65.6%; *P* = .002), and those in poor or fair health (47.5%; 95 CI, 39.0%–56.0%; *P* = 0.03) relative to their counterparts. We found significant differences by education levels (*P* = .02) for men. For women, the prevalence of aspirin use for primary prevention was significantly higher among those in fair or poor health (56.5%; 95% CI, 49.4%–63.6%; *P* = .003) and those with diabetes (60.6%; 95% CI, 53.0%–68.2%; *P* < .001) than among their counterparts. We found significant differences among women by income level (*P* = .02) (Table 1).

**Table 1:** Characteristics of Eligible Mississippi Adults^a^ Taking Aspirin for the Primary Prevention of Cardiovascular Disease, Mississippi Behavioral Risk Factor Surveillance System, 2013

Characteristic	Men Aged 45–79 Years			Women Aged 55–79 Years		
	Weighted Frequency	Weighted % (95% CI)	*P* Value^b^	Weighted Frequency	Weighted % (95% CI)	*P* Value^b^
**Overall**	133,052	39.1 (35.4–42.9)		107,814	45.9 (42.3–49.4)	
**Race**						
Black	36,082	34.0 (27.0–41.1)	.08	35,237	48.5 (42.1–55.0)	.35
White	96,770	41.7 (37.3–46.1)		72,421	44.8 (40.6–49.1)	
**Education**						
<High school diploma	23,225	30.4 (21.8–39.0)	.02	24,285	47.0 (37.4–56.6)	.31
High school diploma or equivalent	41,391	37.6 (31.3–43.9)		39,079	49.2 (43.2–55.2)	
>High school diploma	68,435	44.7 (39.4–50.1)		44,157	42.6 (38.0–47.2)	
**Annual household income, $ **						
<35,000	58,244	41.2 (35.1–47.3)	.57	54,993	48.8 (43.3–54.3)	.02
≥35,000	64,604	38.2 (33.0–43.4)		33,243	39.5 (34.1–45.0)	
No answer	10,204	34.6 (22.6–46.5)		19,638	51.1 (43.4–58.7)	
**Health insurance**						
Yes	115,576	41.2 (37.1–45.4)	.02	95,911	47.6 (44.0–51.2)	.07
No	17,441	29.5 (20.8–38.1)		11,791	35.5 (23.5–47.5)	
**General health**						
Fair/poor	34,164	47.5 (39.0–56.0)	.03	37,690	56.5 (49.4–63.6)	.001
Excellent/good	98,888	36.9 (32.7–41.1)		70,038	41.8 (37.9–45.7)	
**Current smoking**						
Yes	29,336	36.0 (27.9–44.2)	.37	16,809	43.8 (33.6–54.0)	.66
No	103,337	40.3 (36.1–44.6)		90,155	46.2 (42.5–50.0)	
**Body mass index, kg/m^2^**						
<25.0	21,686	31.9 (24.2–39.6)	.12	30,108	45.7 (38.7–52.7)	.27
25.0 to <30.0	56,059	40.5 (34.6–46.4)		33,879	43.3 (37.1–49.5)	
≥30.0	53,892	40.5 (34.6–46.4)		37,453	50.4 (44.5–56.2)	
**Diabetes**						
Yes	29,225	56.0 (46.4–65.6)	.001	27,838	60.6 (53.0–68.2)	<.001
No	103,827	36.1 (32.0–40.2)		79,977	42.3 (38.3–46.3)	

Abbreviation: CI, confidence interval.

a Respondents who had never been told by a physician or health care provider that they had a stroke, heart attack, or coronary heart disease.

b Determined by using χ^2^ test.

Overall, of those with CVD, 85.9% (95% CI, 80.8%–91.0%) of men and 85.1% (95% CI, 79.8%–90.5%) of women were taking aspirin. Among men with CVD, the prevalence of aspirin use was significantly higher among white men (90.2%; 95% CI, 85.8%–94.6%) than among black men (76.0%; 95% CI, 63.1%–89.0%) (*P* = .01). Among women with CVD, aspirin use was significantly higher among those with diabetes (94.4%; 95% CI 89.4%–99.4%; *P* = .004) than among those without diabetes. We found significant differences by education level among women (*P* = .04) (Table 2).

**Table 2:** Eligible Mississippi Adults^a^ Taking Aspirin for the Secondary Prevention of Cardiovascular Disease, Mississippi Behavioral Risk Factor Surveillance System, 2013

Characteristic	Men Aged 45–79 Years			Women Aged 55–79 Years		
	Weighted Frequency	Weighted % (95% CI)	*P* Value^b^	Weighted Frequency	Weighted % (95% CI)	*P* Value^b^
**Overall**	68,563	85.9 (80.8–91.0)		43,100	85.1 (79.8–90.5)	
**Race**						
Black	18,096	76.0 (63.1–89.0)	.01	13,392	81.4 (72.3–90.5)	.36
White	50,331	90.2 (85.8–94.6)		28,911	86.6 (79.8–93.4)	
**Education**						
<High school diploma	22,776	86.4 (76.9–95.9)	.79	12,223	76.2 (63.0–89.3)	.04
High school diploma or equivalent	20,579	88.0 (79.9–96.2)		15,597	88.6 (82.6–94.6)	
>High school diploma	25,208	83.8 (75.4–92.2)		14,972	89.8 (83.2–96.5)	
**Annual household income, $**						
<35,000	40,150	85.4 (78.5–92.5)	.50	25,319	81.6 (73.9–89.2)	.13
≥35,000	22,712	89.0 (81.8–96.2)		6,081	86.9 (73.5–100.0)	
No answer	5,701	78.0 (58.1–97.8)		11,700	92.9 (87.3–98.5)	
**Health insurance**						
Yes	64,666	85.9 (80.7–91.1)	.94	39,134	85.4 (79.8–91.0)	.72
No	3,830	85.1 (62.8–100.0)		3,792	82.0 (62.7–100.0)	
**General health**						
Fair/poor	39,474	86.5 (79.7–93.2)	.96	24,993	84.7 (77.7–91.6)	.85
Excellent/good	28,653	86.7 (79.3–94.2)		18,107	85.8 (77.4–94.1)	
**Current smoking**						
Yes	18,993	82.9 (72.0–93.9)	.49	8,398	76.8 (61.2–92.3)	.13
No	49,569	87.1 (81.5–92.7)		34,408	87.3 (82.2–92.5)	
**Body mass index, kg/m^2^**						
<25.0	12,660	82.8 (71.0–94.6)	.16	10,634	88.3 (81.0–95.7)	.40
25.0 to <30.0	27,070	93.2 (87.8–98.6)		13,946	87.1 (77.4–96.7)	
≥30.0	26,920	85.0 (77.3–92.7)		15,791	80.2 (70.2–90.2)	
**Diabetes**						
Yes	27,073	89.0 (81.9–96.0)	.33	17,976	94.4 (89.4–99.4)	.004
No	41,489	84.0 (77.1–90.8)		24,992	79.4 (71.6–87.3)	

Abbreviation: CI, confidence interval.

a Respondents who reported that a doctor or health care provider had previously told them that they had been diagnosed with a stroke, heart attack, or coronary heart disease.

b Determined by using χ^2^ test.

## Discussion

In 2013, more than a third of eligible Mississippians without CVD were taking aspirin daily for primary prevention, while most (over 85%) of those with CVD were taking aspirin daily. This finding is consistent with results reported for other states (8). In addition, the overall high rate of aspirin use for secondary prevention among eligible Mississippi adults is similar to results reported for nationally representative samples (9). The ongoing collaborative efforts of MSDH and CDC to promote the ABCS of heart disease prevention in the Mississippi Delta region, which has a disproportionately high burden of CVD, must seek to increase the awareness and promotion of strategies that target those with less education, without health insurance, and without diabetes and those with good or excellent health who are eligible for primary prevention. Evidence-based practice and clinical management procedures following CVD events, aspirin-use counseling, and community-based interventions to promote the benefits of regular aspirin use among those eligible are needed to increase aspirin use (9), particularly among black adults with CVD. Future studies should further examine the disparities in aspirin use among eligible Mississippi adults.

These findings have potential limitations. First, the data are self-reported, which could lead to recall bias (9), and aspirin does not require a prescription for purchase. Second, because of the cross-sectional study design there could be temporality bias. Third, the questions used in BRFSS to define CVD might underestimate CVD in the population, because they do not refer to all types of CVD. Increased aspirin use could improve CVD risk prevention and reduction among eligible Mississippi adults. Our findings indicate that a significant proportion of eligible Mississippi adults could benefit from using aspirin to prevent CVD (6).
